# Detailed Investigation of Separation Performance of a MMM for Removal of Higher Hydrocarbons under Varying Operating Conditions

**DOI:** 10.3390/membranes6010016

**Published:** 2016-02-25

**Authors:** Heike Mushardt, Marcus Müller, Sergey Shishatskiy, Jan Wind, Torsten Brinkmann

**Affiliations:** Institute of Polymer Research, Helmholtz-Zentrum Geesthacht, Geesthacht 21502, Germany; heike.mushardt@tuhh.de (H.M.); marcus.mueller86@gmx.de (M.M.); sergey.shishatskiy@hzg.de (S.S.); jan.wind@hzg.de (J.W.)

**Keywords:** mixed matrix membrane, activated carbon, gas permeation, n-butane/methane separation, operating conditions, multi-component mixture

## Abstract

Mixed-matrix membranes (MMMs) are promising candidates to improve the competitiveness of membrane technology against energy-intensive conventional technologies. In this work, MMM composed of poly(octylmethylsiloxane) (POMS) and activated carbon (AC) were investigated with respect to separation of higher hydrocarbons (C_3+_) from permanent gas streams. Membranes were prepared as thin film composite membranes on a technical scale and characterized via scanning electron microscopy (SEM) and permeation measurements with binary mixtures of *n*-C_4_H_10_/CH_4_ under varying operating conditions (feed and permeate pressure, temperature, feed gas composition) to study the influence on separation performance. SEM showed good contact and absence of defects. Lower permeances but higher selectivities were found for MMM compared to pure POMS membrane. Best results were obtained at high average fugacity and activity of *n*-C_4_H_10_ with the highest selectivity estimated to be 36.4 at *n*-C_4_H_10_ permeance of 12 m_N_^3^/(m^2^·h·bar). Results were complemented by permeation of a multi-component mixture resembling a natural gas application, demonstrating the superior performance of MMM.

## 1. Introduction

Today, natural gas is the fastest growing energy source and with a worldwide production of 3.5 billion m_N_^3^ per year making it a highly promising market for separation processes [1]. Around 95% of all industrial separations are covered by the refinery and processing of fossil fuels like crude oil or natural gas or the treatment of associated effluent gas [2]. In this field, the separation of higher hydrocarbons (C_3+_) from permanent gas streams (e.g., CH_4_, N_2_, H_2_) is of great importance. In processing of natural or associated gas, the separation is usually required to result in a consumer-grade product, prevent formation of hydrocarbon condensates during transport that might be harmful for pipeline systems and make use of valuable by-products [3,4]. Separation is mostly performed by conventional methods such as pressure swing adsorption, cryogenic distillation, expansion or absorption, which are usually accompanied by complex process design, high energy demand and high operating costs [4]. Much research has been performed, but despite their highly energy and cost intensive character, only few technologies are competitive today.

A promising alternative are membrane-based separations due to their low energy demand, small plant sizes, environmental friendliness and ease of operation [5]. State-of-the art materials for the separation of condensable hydrocarbons (C_3+_) are siloxane based polymers such as poly(dimethylsiloxane) (PDMS) or poly(octylmethylsiloxane) (POMS) with a highly rubbery character and a solubility controlled permeation in favour of higher hydrocarbons. They have achieved commercial significance in some areas of application today but their performance requires further improvement to ensure competitiveness. Both a high transmembrane flux and a good selectivity are required for a reasonable plant size and energy demand [6]. Further challenges are the harsh conditions regarding pressure, temperature and chemicals that the membrane material has to withstand in natural gas processing operations [7].

The choice of membrane material is crucial for separation. Whereas almost all commercial gas separations are currently based on dense polymeric membranes as they are easy to fabricate as thin film composite membranes on porous support structures, as integral asymmetric hollow fibers or flat sheet membranes while offering good separation behaviour and mechanical strength, conventional polymer chemistry seems to be exploited, and no further revolutionary improvement is expected by just modifying polymeric structure. Porous inorganic membranes made of ceramic, zeolite or carbon can provide superior separation properties, especially when molecular sieving or a selective surface flow can be achieved [8]. They are also beneficial for high temperature applications and harsh chemical conditions [5]. However, despite their superior selectivity and other advantages, widespread industrial application of inorganic membranes is hampered by serious drawbacks regarding their defect-free production, low mechanical strength, brittleness and difficulties with transfer into membrane modules, as well as their costs being up to 10 times higher compared to polymeric membranes [1]. The concept of hybrid mixed-matrix materials (MMMs) based on inorganic filler particles dispersed in a polymeric matrix has attracted much interest and research in recent years. Advantages of both materials are exploited resulting in improved separation performance, mechanical, thermal or chemical stability or specific properties such as conductivity [9]. Achieving this simple but challenging concept faces many difficulties, for example, the careful selection of appropriate materials, their merging into ideal, defect-free structures and transfer of pure material properties into the assembled mixed-matrix structure. Most important for formation of a successful MMM is the interface between continuous polymeric matrix and dispersed filler which directly affects the separation performance [10]. Ideal interface morphology is characterised by a good contact between both materials and shall not show non-selective voids, leaky interface, sieve-in-a-cage morphology, rigidified polymeric layers or blocked pores. Further difficulties are the uniform dispersion of particles within polymeric matrix without agglomeration or sedimentation, realisation of high loadings in thin separation layers and reproducibility of production [11]. So far, no industrial realisation of MMM concepts for gas separation has been reported in literature to our knowledge. Research has been focused mostly on zeolites, carbon molecular sieves (CMS), silica or metal organic frameworks (MOF) as filler materials aiming at molecular sieving and diffusion selectivity meaningful for glassy polymers as matrix for fillers [12,13,14]. While often investigations of MMM performance for O_2_/N_2_ or CO_2_/CH_4_ separations can be found in literature, only few reports deal with the preferential separation of large, condensable hydrocarbons such as *n*-C_4_H_10_ from permanent CH_4_ gas streams.Jia *et al.* [15] studied the permeation of various gases through silicalite filled PDMS membranes. They observed a change of selectivity due to the sieving properties of silicalite which facilitated the permeation of small molecules and impeded the usually high permeation of large, condensable components. In further studies, mostly silica has been applied as filler material in both rubbery and glassy matrix materials for this purpose [16,17,18]. Khanbabaei *et al.* [16] reported a decreased sorption capacity for PDMS filled with fumed silica and different optimums in filler loading with respect to permeability and selectivity. The work of Nunes *et al.* [17] describes MMMs prepared via sol-gel approach with PDMS and tetraethyl orthosilicate (TEOS). While *n*-C_4_H_10_/CH_4_ selectivity could be increased 10-fold for 14% TEOS due to reduced swelling, scattered values for permeability highlighted the difficulties in production of uniform layers. The authors further emphasized the importance of mixed gas experiments to account for coupling or swelling effects. Merkel *et al.* [18] reported an increase of *n*-C_4_H_10_permeability for addition of silica to glassy PTMSP. No improvement of selectivity was found due to the large free volume that allows the accommodation of filler particles without remarkable effect on polymeric structure but with a reduced tendency for plasticization or swelling. An improvement of both permeability and *n*-C_4_H_10_/CH_4_ mixed gas selectivity (21 for 45 wt% silica at 25 °C) has been reported by He *et al.* for silica filled PMP resulting from disrupted chain packing and effective blocking of CH_4_ diffusion by condensed *n*-C_4_H_10_ in free volume [19].

The promising application of MMMs based on POMS and activated carbon (AC) has been described in our previous work [20,21]. Best performance was found for a filler content of 20 wt% showing an improved selectivity *n*-C_4_H_10_/CH_4_ up to 20% compared to pure POMS. Based on these results, the preparation technique has been further optimized and successfully transferred into a large scale production of up to 100 m^2^ membrane area. The performance of MMMs is determined by an interplay of structural parameters, for example the type and shape of filler particles, the filler content, the properties of the polymeric matrix, as well as process conditions [12]. Its good performance shall further be highlighted in this work regarding the influence of a multitude of operating conditions to identify the best suited application range.

## 2. Results and Discussion

### 2.1. SEM Analysis

**Figure 1:**
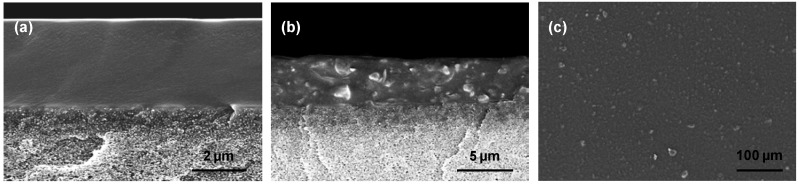
SEM images of pure POMS (**a**) and MMM (**b**) cross-sections of separation layers and (**c**) surface of MMM composed of POMS and 20 wt% activated carbon (d_50_= 1.5 µm).

Figure 1 shows SEM images of cross-section and surface of a TFC MMM with 20 wt% AC. A good contact between polymeric and inorganic material and a quite uniform dispersion of particles is revealed. The addition of particles results in a slight increase of separation layer thickness giving values of 3.9 µm for MMM compared to 3.4 µm of pure TFC POMS membranes. Reported values are averages of at least two different samples. No visible defect formation indicating improper material selection was observed.

### 2.2. Single Gas Permeation

**Figure 2:**
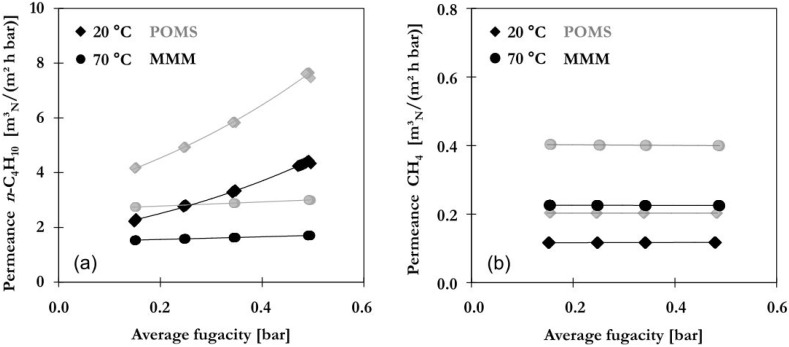
Permeance of (**a**) *n*-C_4_H_10_ and (**b**) CH_4_ at different average fugacities in POMS (**grey**) and MMM filled with 20 wt% AC (**black**) determined with pure gases at different temperatures (lines represent exponential trend curves).

Single gas measurements depict an idealized membrane performance as no coupling or competitive effects between gas mixture components occur. Nonetheless, such investigation is necessary to understand fundamental differences in transport through the membrane between gas components. Results for permeation of single gases are presented in Figure 2 in terms of *n*-C_4_H_10_ and CH_4_permeances as function of average fugacity at 20 or 70 °C for POMS (grey) and MMM filled with 20 wt% AC (black). The average fugacity is estimated as average between fugacities applied on feed and permeate side of membrane and used as reference for the corresponding concentration of gas components within the membrane [22].

The permeance of *n*-C_4_H_10_ (Figure 2a) shows an increase with feed pressure or average fugacity in POMS and MMM with the exponential trend curve indicated by the solid lines. Higher values are found for POMS resulting from slightly lower thickness of separation layer. The tortuosity created by addition of AC is expected to slow down diffusive transport. Permeation is controlled by solubility with both solution of *n*-C_4_H_10_ into polymeric matrix and adsorption on AC being dependent on pressure. Dissolution of condensable *n*-C_4_H_10_ into polymeric matrix causes a loosening of polymeric chains known as swelling and thus higher diffusive flux [23]. This is especially pronounced at lower temperatures as permeation is highly controlled by solubility with lower temperatures being beneficial for solubility and adsorption. No pressure dependency was found for CH_4_ permeance in POMS or MMM (see Figure 2b). Constant values are found for each temperature between 20 and 70 °C with values ranging from 0.2 to 0.4 m_N_^3^/(m^2^·h·bar) for POMS and 0.12 to 0.23 m_N_^3^/(m^2^·h·bar) for MMM. Permeation of non-condensable components is dominated by diffusion and facilitated by higher temperatures due to enhanced mobility of gas molecules. The ideal selectivity was calculated from *n*-C_4_H_10_ permeances extrapolated to zero feed pressure. No significant difference was found between POMS and MMM. Ideal selectivity was estimated to be 15.6 (POMS) and 14.6 (MMM) at 20 °C and 6.6 (POMS) and 6.5 (MMM) at 70 °C with the decrease resulting from lower *n*-C_4_H_10_ permeation.

### 2.3. Binary Gas Mixture Permeation and Separation

As stated above, single gas measurements are not sufficient to correctly evaluate separation performance due to neglect of the coupling effects present in real gas mixtures. For this reason, the performance of MMM has been investigated with binary gas mixtures *n*-C_4_H_10_/CH_4_ under a variety of operating conditions [24].

#### 2.3.1. Influence of Feed Pressure

A significant influence of feed pressure is expected as it is directly affecting the driving force for permeation. Measurements have been performed with feed pressures ranging from 10 to 40 bar at a constant temperature of 20 °C of feed gas and a permeate pressure of 1.2–1.6 bar.

**Figure 3:**
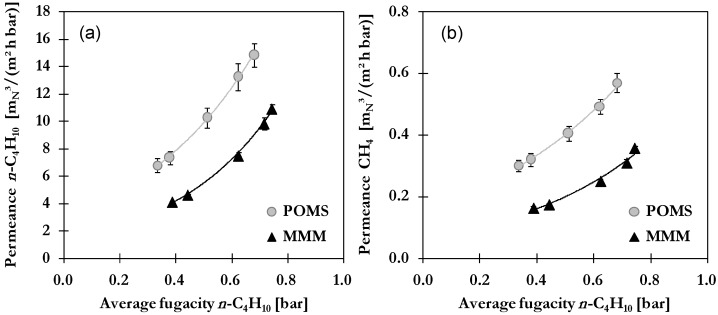
Influence of average *n*-C_4_H_10_ fugacity on permeance of (**a**) *n*-C_4_H_10_ and (**b**) CH_4_ in POMS (**grey**) and MMM with 20 wt% AC (**black**) for a binary feed mixture with 5 mol% *n*-C_4_H_10_ at 20 °C (lines represent exponential trend curves).

Results for *n*-C_4_H_10_ and CH_4_ permeances as a function of average fugacity *n*-C_4_H_10_ resulting from variation of feed pressure are presented in Figure 3 with lines representing exponential trend curves. For both membrane types as well as gas components, an increase of permeance is shown. While no great differences can be observed between trends in both cases in POMS and MMM, the increase is much more pronounced for *n*-C_4_H_10_ compared to CH_4_. As described for single gas *n*-C_4_H_10_, the increase is caused by the dissolution and swelling of polymeric matrix supported by the enhanced adsorption on activated carbon in case of MMM. For example, the permeance *n*-C_4_H_10_ shows an increase from 6.8 to 14.8 m_N_^3^/(m^2^·h·bar) in POMS and from 4.1 to 10.9 m_N_^3^/(m^3^·h·bar) in MMM. While no pressure dependency was found for single gas CH_4_ permeance, an increase can be observed in case of binary mixture. The CH_4_ permeation is directly coupled to the concentration of dissolved *n*-C_4_H_10_ which not only facilitates CH_4_ diffusion by polymer swelling but also creates a more favorable environment for solution of CH_4_ [25]. The degree of swelling can be evaluated from the dependence of hydrocarbon permeation on the average fugacity of *n*-C_4_H_10_ and results are thus presented in Figure 3b. The CH_4_permeance increases from 0.3 to 0.6 m_N_^3^/(m^2^·h·bar) in POMS and 0.16 to 0.36 m_N_^3^/(m^2^·h·bar) in MMM. A lower degree of swelling is expected for the MMM as part of the *n*-C_4_H_10_ is bound to adsorption sites of activated carbon and not available for dissolution into polymeric matrix. In addition, the presence of particles impairs the mobility of polymeric chains and thus reduces the ability to loosen up the structure [17,18].

**Figure 4:**
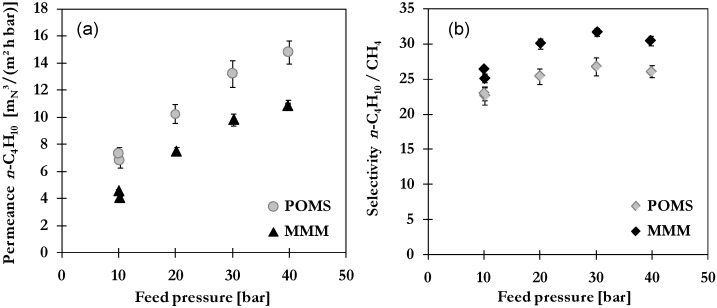
Influence of feed pressure on (**a**) permeance of *n*-C_4_H_10_ and (**b**) *n*-C_4_H_10_/CH_4_ selectivity of POMS (**grey**) and MMM with 20 wt% AC (**black**) for a binary feed mixture with 5 mol% *n*-C_4_H_10_ at 20 °C.

The influence of feed pressure on permeance *n*-C_4_H_10_ and selectivity *n*-C_4_H_10_/CH_4_ is illustrated in Figure 4b. The same trends are observed for POMS and MMM with increasing selectivity up to 30 bar followed by a slight decrease or flattening. Nonetheless, a higher selectivity is found for MMM over the whole investigated pressure range with best results at 30 bar. This improvement is attributed to the high affinity of *n*-C_4_H_10_ toward AC. A high amount of *n*-C_4_H_10_ inside the pores might not only reduce swelling and thus minimize undesired co-permeation but also evoke a selective surface flow and blocking of CH_4_ diffusion via formation of a condensed layer. The slight decrease above 30 bar is caused by excessive swelling of polymeric matrix and increased competition among gas components as transport through swollen polymer is to a greater degree governed by diffusion which favors smaller CH_4_ molecules. In case of a severely swollen, highly permeable matrix, this might even cause a by-passing of filler particles. Furthermore, a stronger competition between *n*-C_4_H_10_ and CH_4_ for adsorption sites in activated carbon is likely at high pressure. The thermodynamic selectivity of adsorbent materials is often reported to level off at high pressures due to co-adsorption of the lower affinity component supporting the decrease of MMM selectivity curves at higher pressure.

#### 2.3.2. Influence of Permeate Pressure

**Figure 5:**
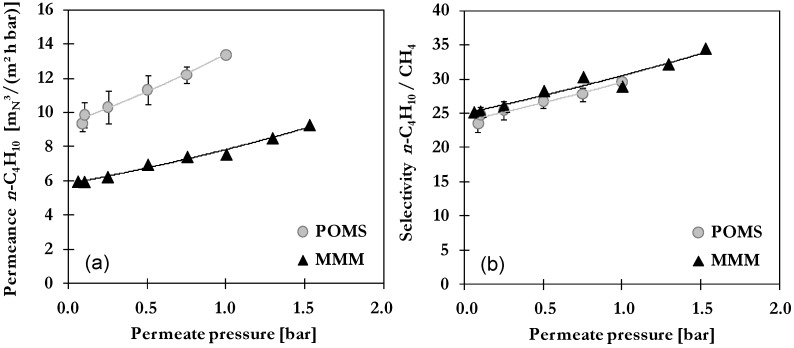
Influence of permeate pressure on (**a**) permeance of *n*-C_4_H_10_ and (**b**) *n*-C_4_H_10_/CH_4_ selectivity of POMS (**grey**) and MMM with 20 wt% AC (**black**) for a binary feed mixture with 5 mol% *n*-C_4_H_10_ at 30 bar and 20 °C (lines represent exponential trend curves).

In order to increase the driving force for permeation and also the pressure-ratio influencing the separation performance, membrane-based separations are often performed with vacuum on the permeate side [5]. Thus, the influence of permeate pressure was investigated by installing a vacuum pump and adjusting values between 0.05 and 1.5 bar. Meanwhile, the feed pressure and temperature were kept constant at 30 bar and 20 °C. Results are presented in Figure 5 in terms of permeances for *n*-C_4_H_10_ (a) and selectivity *n*-C_4_H_10_/CH_4_ (b) as function of permeate pressure with lines representing exponential trend curves.

Again, similar trends are found for POMS and MMM. Both show decreasing permeances of *n*-C_4_H_10_ and CH_4_ with lowering of the permeate pressure. Contrary effects are evoked by variation of permeate pressure. On the one hand, a decrease of permeate pressure at constant feed pressure allows a higher pressure ratio across the membrane. The higher this ratio, the higher the driving force for permeation which especially favors the diffusion of small molecules such as CH_4_. This enhancement seems to be superimposed by the reduced solubility at lower pressure or associated lower average fugacity respective to the degree of swelling. By comparing the two membrane types, the effect is more pronounced for POMS as indicated by the higher slope of trend curve (0.36 for POMS and 0.29 for MMM). The selectivity shows the same decreasing trend in relation to decreasing permeate pressure as permeances in both POMS and MMM (see Figure 5b). Results are in the range of 21.6–27.2 for POMS and 25.1–30.3 for MMM. Best performance could thus be achieved at higher permeate pressure. This is quite beneficial as additional energy- or cost intensive application of vacuum pumps can be avoided at least in the case of feed pressure greater than 10 bar.

#### 2.3.3. Influence of Temperature

**Figure 6:**
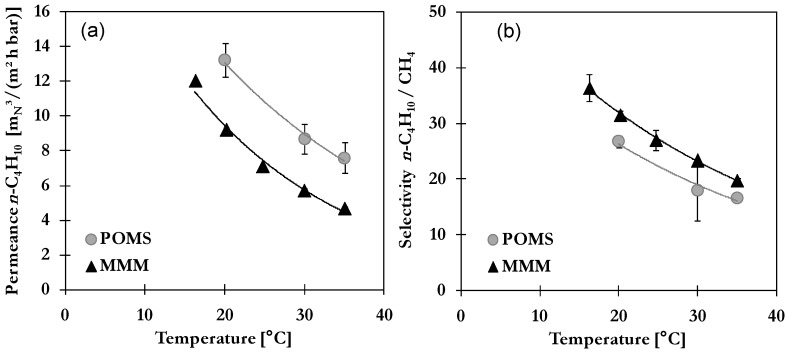
Influence of temperature on (**a**) *n*-C_4_H_10_ permeance and (**b**) *n*-C_4_H_10_/CH_4_ selectivity of POMS (**grey**) and MMM with 20 wt% AC (**black**) for a binary feed mixture with 5 mol% *n*-C_4_H_10_ at 30 bar (lines represent exponential trend curves).

As stated above, the temperature has a significant influence on permeation as it impacts solubility in POMS, adsorption on AC and diffusion. The mixed gas permeation and separation as a function of temperature is presented in Figure 6 for a constant feed pressure of 30 bar. Similar trends are observed for POMS and MMM with both showing solubility controlled permeation over the investigated temperature range. The permeance of *n*-C_4_H_10_ (Figure 6a) decreases with increasing temperature reflecting the lower *n*-C_4_H_10_ solubility respective to the lower degree of swelling of the selective layer. Values for MMM are estimated to be in the range of 12–4.7 m_N_^3^/(m^2^·h·bar) for 15–35 °C. The dissolution of gas into the polymeric matrix is a two-step mechanism composed of condensation and mixing. At higher temperatures, *n*-C_4_H_10_ exhibits a lower activity in gas phase which in turns affects its readiness to condense and thus decreases solubility [25]. This is in good agreement with behaviour of single gas permeation (see Figure 2a). For CH_4_ a decrease of mixed gas permeation from 0.33 to 0.24 m_N_^3^/(m^3^·h·bar) in MMM with increasing temperature was found which is in strong contrast to single gas behaviour. The enhanced diffusivity seems to be compensated by decreased swelling in presence of *n*-C_4_H_10_. In addition, transport through AC is still in favour for *n*-C_4_H_10_. The influence of temperature on permeation results in a decrease of mixed gas selectivity for both studied membranes as depicted in Figure 6b. The selectivity *n*-C_4_H_10_/CH_4_ decreased from 26.8 to 16.7 for POMS and from 31.6 to 19.8 for MMM in the temperature range of 20–35 °C. For a successful application of both POMS and MMM, lower temperatures should be selected that ensure a high solubility in the polymeric matrix as well as high amount of adsorbed *n*-C_4_H_10_ in the pore system of activated carbon.

#### 2.3.4. Influence of Binary Feed Composition

**Figure 7:**
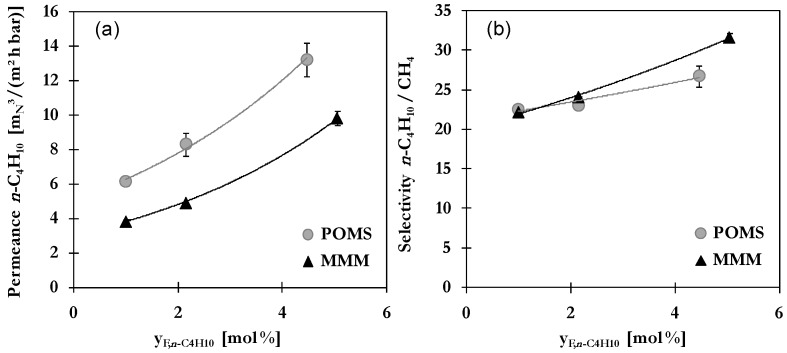
Influence of binary mixture composition on (**a**) *n*-C_4_H_10_ permeance and (**b**) *n*-C_4_H_10_/CH_4_ selectivity of POMS (**grey**) and MMM with 20 wt% AC (**black**) at 20 °C and 30 bar (lines represent exponential trend curves).

Industrial separations typically involve integral changes of compositions in the separation system, *i.e.*, compositions are changing substantially between the feed and permeate sides of a membrane module. Furthermore, fluctuating compositions of feed mixtures to be separated impose a great challenge for separation process. A low concentration of desired component usually makes it more difficult to separate as low driving forces are available while high concentrations can cause excessive swelling or even degradation of polymeric matrix in case of corrosive components. For hydrocarbon mixtures, the higher hydrocarbons are usually present only in minor amounts. To evaluate membrane performance, even under these unfavourable conditions, measurements were performed with binary mixtures containing 1, 2 or 5 vol-% *n*-C_4_H_10_. Permeance of *n*-C_4_H_10_ in POMS and MMM and the *n*-C_4_H_10_/CH_4_ selectivity as function of binary mixture composition are presented in Figure 7 for 20 °C and 30 bar.

The permeance shows an exponential increase with feed concentration of *n*-C_4_H_10_. This again can be related to the average fugacity of *n*-C_4_H_10_ and associated degree of swelling and adsorption. As was to be expected, more gas can dissolve or adsorb if it is present in higher concentrations in feed mixture causing an increase of average fugacity. Comparison of slopes reveals a similar increase for *n*-C_4_H_10_ in POMS (0.22) and MMM (0.23) but a lower increase for CH_4_ in MMM (0.14) compared to POMS (0.17). It is assumed that less CH_4_ can permeate due to a reduced swelling of polymer as well as a dominating *n*-C_4_H_10_ phase in pore system blocking pathways for CH_4_ diffusion. The influence of feed composition on selectivity is given in Figure 7b. At low concentrations of 1 or 2 vol-% *n*-C_4_H_10_, both membranes show nearly the same selectivity but the beneficial effect of AC particles is clearly demonstrated in terms of the improved selectivity at higher *n*-C_4_H_10_ concentrations. Feed mixtures with more than 2 vol-% *n*-C_4_H_10_ are thus recommended for the application of MMM based on POMS and AC.

### 2.4. Multi-Component Gas Mixture Permeation and Separation

The improved binary separation performance of the MMM should be further evidenced for the case of a multi-component mixture resembling a typical natural gas application. The mixture is composed of alkanes from CH_4_ up to *n*-C_5_H_12_ and CO_2_. The total concentration of higher hydrocarbons (C_3+_) was approximately 19 vol-%. Measurements have been performed at feed pressures between 10 and 40 bar and a constant temperature of 20 °C.

Permeances of components estimated at 20 °C and 40 bar feed pressure are summarized in Table 1.

**Figure 8:**
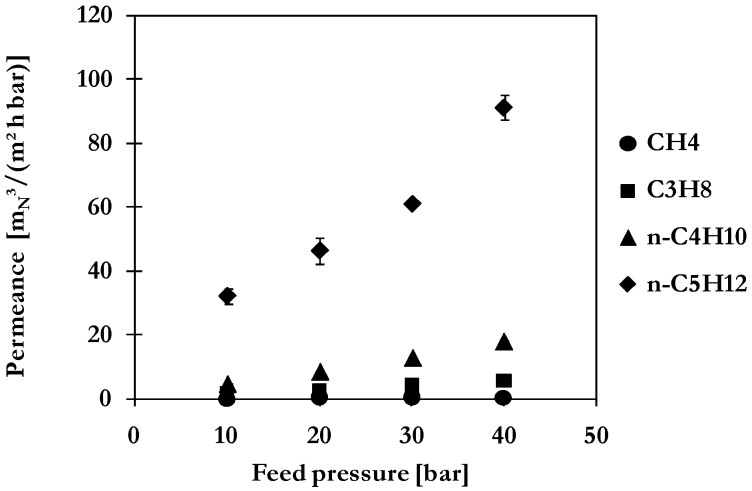
Permeances of selected hydrocarbon components in multi-component mixture separation with MMM composed of POMS and 20 wt% AC at 20 °C (mixture composition: 1 vol-% *n*-C_5_H_12_, 2 vol-% *n*-C_4_H_10_, 6 vol-% C_3_H_8_, 10 vol-% C_2_H_6_, 0.79 vol% CH_4_ and 2 vol-% CO_2_).

The order of permeance and selectivity values follows the condensability of penetrants indicated by the boiling point (see Table 1) which increases with number of carbon atoms for hydrocarbon components. The lowest permeance is thus observed for non-condensable CH_4_ followed by CO_2_, C_2_H_6_, C_3_H_8_ and *n*-C_4_H_10_. By far the highest permeance arises for *n*-C_5_H_12_ whose boiling point of 36 °C [22] indicates the great willingness to condense. All components show an increase of permeance with increasing feed pressure or, respectively, the swelling of polymeric matrix, which is illustrated for selected components in Figure 8.

**Figure 9:**
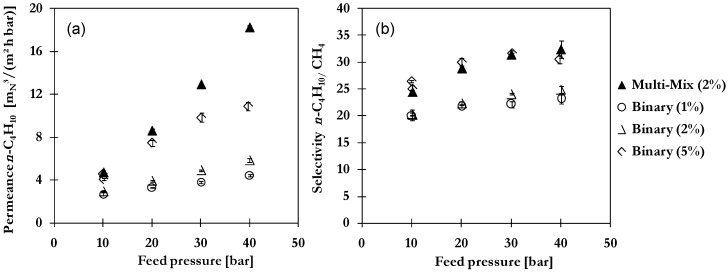
Comparison of (**a**) *n*-C_4_H_10_ permeance and (**b**) *n*-C_4_H_10_/CH_4_ selectivity of MMM in multi-component mixture separation (2 vol-% *n*-C_4_H_10_) and binary mixture separation (1, 2 or 5 vol-% *n*-C_4_H_10_) at 20 °C.

The selectivity with respect to CH_4_ shows the same trend and increases with increasing condensability. Higher selectivities for each gas component are achieved by application of MMM instead of pure POMS with greatest improvement for *n*-C_4_H_10_ (13%) and *n*-C_5_H_12_ (42%) resulting from the adsorptive capacity and reduced tendency in case of activated carbon. By comparison of binary and multi-component mixture separation results, a greater increase of permeation (Figure 9a) with pressure is observed due to the presence of *n*-C_5_H_12_. This enhancement results in a higher selectivity compared to values achieved with binary mixtures of same concentration *n*-C_4_H_10_ in feed as illustrated in Figure 9b. No negative impacts due to coupling or competitive mixture effects were noticed. The enhanced separation performance of MMM was clearly demonstrated even for multi-component mixtures.

## 3. Experimental Section

### 3.1. Materials

Membranes are composed of poly(octylmethylsiloxane) (POMS) as polymeric matrix. POMS is a member of the siloxane polymer family which includes the well-known poly(dimethylsiloxane) (PDMS), the industrial state-of-art material for separation of higher hydrocarbons [17]. POMS has a highly rubbery character indicated by a glass transition temperature of −110 °C giving a solubility selective permeation. A precursor solution for membrane casting was prepared in iso-octane (Merck KGaA, purity >99.5%) as solvent with a number of siloxane based cross-linking and reinforcement agents and a platinum-based catalyst to initialise cross-linking reaction. Since POMS membranes are produced and employed on commercial basis, the exact composition of precursor solution cannot be disclosed. Microporous activated carbon (AC) was used as inorganic filler phase. It was provided by Blücher GmbH as sieving fraction with mean particle size of 1.5 µm. Further characteristic properties are summarized in Table 2. The AC was selected due to its high affinity towards C_3+_ hydrocarbons with the adsorption isotherm following the equation of Tóth [20]. AC particles were dried at 150 °C in a vacuum oven for at least 24 h prior to use. All gases were purchased at Linde (purity of 99.5%), liquid *n*-C_5_H_12_ (>99.9%) at Merck KGaA and were used as received.

### 3.2. Membrane Preparation

To facilitate dispersion, dried AC particles were preliminarily blended with isooctane and sonicated for 40 min using an ultrasonic bath by Elmasonic S30H. MMM coating suspensions were prepared by stepwise addition of polymer precursor solution and catalyst to isooctane saturated AC particles and thoroughly mixing with a dissolver for 10–20 min at 8000 rpm (Dispermat^®^, VMA Getzmann GmbH) after each step. The AC content was adjusted to 20 wt% with respect to mass of polymer as identified as optimum filler content in previous work [20]. A final 2 min sonication step was applied prior to casting to ensure homogeneous dispersion and removal of trapped air. Pure POMS thin film composite (TFC) membranes have been prepared as reference material. Membranes have been prepared as TFC membranes by suspension coating on a PDMS coated support structure of microporous polyacrylonitrile (PAN) on non-woven polyester (PE). A roll-coating machine was used for TFC membrane production. An integrated oven situated immediately after the coating module allows a thermal treatment at 100 °C for 5 min to induce cross-linking and solvent removal. A final PDMS layer (0.5 wt% PDMS in iso-octane) was deposited on top of the selective mixed matrix layer to eventually seal formed surface defects.

### 3.3. SEM Analysis

MMM morphology was analysed via scanning electron microscopy (SEM) images of membrane surfaces and cross-sectional areas. Dried samples were immersed in isopropanol, freeze-fractured in liquid nitrogen and coated with a 2 nm layer of platinum. Images were taken with SEM system LEO 1550 VP by Zeiss.

### 3.4. Pure Gas Permeation Measurements

Permeation measurements were performed with pure *n*-C_4_H_10_ and CH_4_ at feed pressures up to 1 bar in the temperature range of 20–70 °C. An automated set-up operating in constant volume and variable pressure mode was used to determine permeance via time dependent change of pressure in feed and permeate vessels. The set-up is described elsewhere in more detail [27,28].

### 3.5. Mixed Gas Permeation Measurements

Prior to characterisation, all samples were dried in a vacuum oven at 80 °C and 7 mbar for at least 15 h to ensure the complete removal of residual solvent and allow activation of filler particles. The experimental mixed gas membrane characterisation set-up is depicted elsewhere [20,27]. Membrane samples of 47 mm in diameter were inserted and sealed in the test cell and the system evacuated for at least 45 min. A preliminary prepared gas mixture was filled in a feed vessel and pressurised via a compressor while a gas circulator generates a sufficient flow to avoid concentration polarization. This is ensured by a low stage-cut of less than 1%. The feed mixture was temperature controlled with a water bath before entering the membrane test cell. After equilibrating, the pressure and flow rates of feed, permeate and retentate were measured and the gas composition on feed, permeate and retentate sides analysed with a gas chromatograph (Varian 3400) equipped with a packed metal column (Chromosorb 107, carrier gas argon or helium).

Mixed gas measurements were performed with previously prepared binary mixtures of *n*-C_4_H_10_ in CH_4_ with 1–5 vol-% *n*-C_4_H_10_ in feed under varying operating conditions of 15–35 °C, 10–40 bar feed pressure and 0.05–1.6 bar permeate pressure [24]. A multi-component mixture with composition resembling natural gas has been prepared with 1 vol-% *n*-C_5_H_12_, 2 vol-% *n*-C_4_H_10_, 6 vol-% C_3_H_8_, 10 vol-% C_2_H_6_, 0.79 vol% CH_4_ and 2 vol-% CO_2_ and investigated at 20 °C, feed pressure 10–40 bar and permeate pressure 1.2–1.5 bar. Results are presented as average values for at least two different samples with errors estimated according to the t-distribution.

Since condensable gases like *n*-C_4_H_10_ or *n*-C_5_H_12_ show significant gas phase non-idealities, permeances were calculated based on fugacities (*f_i_*) to account for the real gas behaviour. The fugacity coefficients were estimated by the Soave-Redlich-Kwong (SRK) equation of state. The permeance (*L_i_*) of a component *i* was calculated as the permeate flow rate (*V_i_*) at standard conditions (1.01325 bar, 0 °C) divided by its driving force, namely the fugacity difference between feed and permeate sides, and membrane area (*A_m_*).

(1) $$L_{i}=\frac{{\dot{V}}_{i}}{A_{m}\cdot \left(f_{i,F}-f_{i,P)}\right)}$$

The selectivity given by the ratio of permeances of two components i and j quantifies the separation efficiency. It is always determined with respect to the less permeable component.

(2) $$\alpha_{i/j}=\frac{L_{i}}{L_{j}}$$

## 4. Conclusions

In this work, the performance of a MMM composed of rubbery POMS and 20 wt% AC has been evaluated with respect to separation of higher hydrocarbon from permanent gas streams such as *n*-C_4_H_10_/CH_4_. A solubility selective permeation was found similar to pure POMS. The detailed study of separation performance for binary feed mixtures under varying operating conditions has revealed the superior performance of MMM as well as some guidelines regarding the selection of appropriate application. Best performance was achieved at highest average fugacity of *n*-C_4_H_10_ caused by increasing feed pressure, high permeate pressure and high concentration of condensable hydrocarbon component in feed mixture. Further, low temperatures provide a high activity of *n*-C_4_H_10_. The highest selectivity was estimated to be 36.4 at *n*-C_4_H_10_ permeance of 12 m_N_^3^/(m^2^·h·bar). The superior performance of MMM was also confirmed in experiments on separation of a multi-component mixture similar to natural gas with even higher selectivity *n*-C_4_H_10_/CH_4_ compared to binary mixture separation.

## Figures and Tables

**Table 1:** Boiling point T_b_ [26], permeance L and selectivity *vs.* CH_4_ in multi-component mixture permeation experiments with MMM composed of POMS and 20 wt% AC at 30 bar feed pressure and 20 °C.

Component	CH_4_	CO_2_	C_2_H_6_	C_3_H_8_	*n*-C_4_H_10_	*n*-C_5_H_12_
T_b_ (°C)	−161.5	−78.5	−88.7	−42.1	−0.5	36.0
L (m_N_^3^/(m^2^·h·bar))	0.41	1.20	1.64	4.14	12.95	60.97
Selectivity *vs.* CH_4_	-	2.92	3.98	10.06	31.46	148.12

**Table 2:** Characteristic properties of activated carbon.

Properties	Value
d_50_ µm	1.5
S_BET_ m^2^/g	1361
d_pore_ Å	18.7
v_pore_ cm^3^/g	0.636
Porosity %	57.6
Density cm^3^/g	0.891
